# Analog peptides of type II collagen can suppress arthritis in HLA-DR4 (DRB1*0401) transgenic mice

**DOI:** 10.1186/ar2043

**Published:** 2006-09-18

**Authors:** Yoshihiko Sakurai, David D Brand, Bo Tang, Edward F Rosloniec, John M Stuart, Andrew H Kang, Linda K Myers

**Affiliations:** 1 Department of Medicine, University of Tennessee, 956 Court Avenue, Memphis, TN 38163, USA; 2 Current address: Department of Surgery, National Hospital Organization, Kanagawa Hospital, 666-1 Ochiai, Hadano-city, Kanagawa 257-8585, Japan; 3 Research Service, Veterans Affairs Medical Center, 1030 Jefferson Ave., Memphis TN 38104, USA; 4 Department of Pediatrics, University of Tennessee, 956 Court Avenue, Memphis, TN 38163, USA

## Abstract

Rheumatoid arthritis (RA) is an autoimmune disease associated with the recognition of self proteins secluded in diarthrodial joints. We have previously established that mice transgenic for the human DR genes associated with RA are susceptible to collagen-induced arthritis (CIA) and we have identified a determinant of type II collagen (CII_263–270_) that triggers T-cell immune responses in these mice. We have also determined that an analog of CII_263–270 _would suppress disease in DR1 transgenic mice. Because the immunodominant determinant is the same for both DR1 transgenic and DR4 transgenic mice, we attempted to determine whether the analog peptide that was suppressive in DR1 transgenic mice would also be effective in suppressing CIA in DR4 transgenic mice. We treated DR4 transgenic mice with two analog peptides of CII that contained substitutions in the core of the immunodominant determinant: CII_256–276 _(F263N, E266D) and CII_256–270 _(F263N, E266A). Mice were observed for CIA, and T-cell proliferative responses were determined. Either peptide administered at the time of immunization with CII significantly downregulated arthritis. Binding studies demonstrated that replacement of the phenylalanine residue in position 263 of the CII peptide with asparagine significantly decreased the affinity of the peptide for the DR4 molecule. In contrast, replacement of the glutamic acid residue in position 266 with aspartic acid or with alanine had differing results. Aspartic acid reduced the affinity (35-fold) whereas alanine did not. Both peptides were capable of suppressing CIA. With the use of either peptide, CII_256–276 _(F263N, E266D) or CII_256–270 _(F263N, E266A), the modulation of CIA was associated with an increase in T-cell secretion of IL-4 together with a decrease in IFN-γ. We have identified two analog peptides that are potent suppressors of CIA in DR4 transgenic mice. These experiments represent the first description of an analog peptide of CII recognized by T cells in the context of HLA-DR4 that can suppress autoimmune arthritis.

## Introduction

Susceptibility to rheumatoid arthritis (RA) is strongly associated with the expression of specific HLA class II alleles, especially HLA-DR1 and DR4 [1,2]. It is now known that in most instances this increased susceptibility is associated with the DRB1 locus, and specifically with the presence of DRB1*0101, DRB1*0401, DRB1*0404 or DRB1*0405 allotypes [3]. Type II collagen (CII) has received considerable attention as a candidate autoantigen because it is the predominant protein of articular cartilage, and autoimmunity to CII is commonly detected in patients with RA [4-8]. In support of the role of autoimmunity to CII in RA, we have shown that mice transgenic for the HLA-DR1 (DRB1*0101) or DR4 (DRB1*0401) genes develop collagen-induced arthritis (CIA) after immunization with human CII [9,10]. These data are significant in that they demonstrate a direct link between RA susceptibility alleles and the autoimmunity to CII observed in RA.

Using T cells from the transgenic mice, we have identified CII_263–270 _(FKGEQGPK) as the core of the immunodominant T-cell determinant presented by both HLA-DR1 (DRB1*0101) and DR4 (DRB1*0401) [11]. Previously, we identified a synthetic analog peptide of human CII, CII_256–276 _(F263N, E266D), also called A12, which could profoundly suppress arthritis when administered to DR1 transgenic mice [12]. Considering that both DR1 and DR4 transgenic mice react with a common core epitope, it was of interest to determine whether the analog peptide A12 could also suppress arthritis in the context of DRB1*0401. For these experiments we used a panel of analog peptides of the immunodominant determinant of CII and were able to identify two analog peptides that suppress arthritis in DR4 mice. These peptides included A12 and CII_256–276 _(F263N, E266A), also called A13. To clarify the structural characteristics of the DR4-peptide complexes, we analyzed the DR4-restricted presentation of analogs of the CII_256–276 _peptide with the use of peptide binding assays. These studies reveal that residues 263 and 266 are crucial for interaction with the DR4 molecule. With either A12 or A13, the modulation of CIA was associated with an increase in T-cell secretion of the T helper type 2 cytokine IL-4 together with a decrease in the T helper type 1 cytokine IFN-γ. These experiments represent the first description of analog peptides of type II collagen recognized by T cells in the context of the human DR4 molecule that can suppress autoimmune arthritis.

## Materials and methods

### Animals

B10.M-DR4^+/- ^transgenic mice were raised in our animal facility in a specific pathogen-free environment. The DR4 transgene was constructed as a chimeric molecule composed of DR4 α1 and β1 domains and I-Eα2 and β2 domains. The production of the chimeric genes and the B10.M-DR4 transgenic mouse has been described previously [13]. The B10.M-DR4 mice were maintained as heterozygotes for the DR4 transgene, because homozygous B10.M-DR4 mice from this founder do not survive.

### Immunization

CII was dissolved in 0.01 M acetic acid and emulsified with an equal volume of complete Freund's adjuvant (CFA) as described previously [14]. The resulting emulsion was injected subcutaneously into the base of the tail. Each mouse received a total volume of 0.05 ml containing 100 μg of *Mycobacterium tuberculosis *and 100 μg of antigen. All work was approved by the Institutional Animal Care and Use Committee.

### Preparation of CII

Native CII was solubilized from bovine articular cartilage by limited pepsin digestion and purified as described previously [14].

### Synthesis of analog peptides of CII

The peptide representing the immunodominant determinant of CII and its analog peptides containing specific amino acid substitutions were chemically synthesized by a solid-phase procedure described previously, using a model 430 peptide synthesizer (Applied Biosystems, Foster City, CA, USA) and purified by high-performance liquid chromatography [14].

### Measurement of T-cell cytokines by ELISA

Quantitative measurement of cytokines was performed as described previously [12]. In brief, spleens and lymph nodes from DR4 mice immunized with bovine CII (or CII plus other peptides) emulsified with CFA 10 to 14 days previously were individually minced into single-cell suspensions in Hanks balanced salt solution (HBSS) and washed three times with HBSS. Pooled splenocytes and lymph-node cells were then adjusted to a concentration of 5 × 10^6^/ml and cultured with 100 μg/ml antigen in Dulbecco's modified Eagle's medium (Gibco, Grand Island, NY, USA) supplemented with 5% fetal bovine serum (Hyclone, Logan, UT, USA). Supernatants were collected 72 to 120 hours later and used either fresh or frozen at -70°C. Commercially available kits for IFN-γ (Gibco BRL, Gaithersburg, MD, USA), IL-4 and IL-10 (Endogen, Cambridge, MA, USA) were used for quantitative measurement of murine cytokines.

### Proliferation assays

Mice were immunized subcutaneously with 100 μg of type II collagen emulsified in an equal volume of CFA. Ten days after immunization, draining lymph nodes were removed, disassociated, and washed in HL-1 medium (Bio-Whitaker, Walkersville, MD, USA). Lymphocytes were cultured at 37°C for 4 days with various collagen peptides in 96-well plates at 4.5 × 10^5 ^per well in 300 μl of HL-1 medium supplemented with 50 μM 2-mercaptoethanol and 0.1% BSA in 5% humidified CO_2_. Eighteen hours before the termination of the cultures, 1 μCi of [^3^H]thymidine (New England Nuclear, Boston, MA, USA) was added to each well. Cells were harvested onto glass fiber filters and were counted on a Matrix 96 direct ionization β-counter (Packard Instrument Company, Meriden, CT, USA).

### Purification of DR molecules

Soluble DR1 and DR4 were purified from culture supernatants of transfected S2 *Drosophila *cells as described previously [11]. In brief, the cytoplasmic and transmembrane portions of these molecules were deleted from the cDNA by polymerase chain reaction, a new stop codon was inserted immediately before the transmembrane domain, and the resulting cDNA was cloned into the *Drosophila *expression vector pRmHA-3. S2 cells were transfected with a 10:1 ratio of DRB1 and DRA1 to pUChsneo by precipitation with calcium phosphate. Soluble DR production was induced by 1 mM CuSO_4_, and 5 days later the culture supernatant was collected and adjusted to 0.05% octyl glucoside (OcG). The soluble DR was purified by passage of the supernatant over an affinity column coupled with the anti-DR antibody LB 3.1. The column was washed with 0.05% OcG and 0.15 M NaCl in phosphate buffer, pH 7.5, followed by 0.05% OcG and 0.5 M NaCl in phosphate buffer, pH 7.5. The DR was eluted with 100 mM Tris-HCl, 0.5 M NaCl, pH 11.2, and the fractions were immediately neutralized with acetic acid. The recovered DR was concentrated with an Amicon Stirred Cell (Beverly, MA, USA) and quantified by measurement of *A*_280 _and subjection to SDS-PAGE before use.

### Class II binding assays

Class II binding assays were performed as described by Hill and colleagues [15]. In brief, various concentrations of the competitor peptides were incubated for 4 hours at 37°C, with constant concentrations of biotinylated hemagglutinin (HA) (307–319) peptide (0.5 nM for DR1, 5 nM for DR4) and DR molecule (10 nM) in PBS containing OcG. After incubation, DR-peptide complexes were transferred and captured by incubation overnight at 4°C on a 96-well microtiter plate initially coated with LB3.1 (anti-DR) and blocked with BSA. Excess peptide was removed by washing with PBS containing 0.05% Tween 20. The plates were treated with europium-labeled streptavidin (DELFIA) and incubated for 2 hours at 25°C. After being washed, the plates were treated with a chelating enhancement solution (DELFIA), which releases europium from streptavidin and forms a highly fluorescent micellar solution. Fluorescence was quantified with a microplate fluorimeter (DELFIA; LKB/Pharmacia, Uppsala, Sweden). The concentration of CII peptide inhibiting 50% of the binding of HA peptide (IC_50_) was calculated from the linear portion of the curves. IC_50 _values are the average of two determinations per peptide.

## Results

### T-cell responses to analog peptides

We have previously determined that both DR1 and DR4 mice are susceptible to CIA and respond to the same core determinants in CII. However, T-cell hybridomas generated from each strain are restricted by the respective DR molecules. Hybridomas from DR1 mice do not recognize the core determinant presented by DR4 and vice versa [11].

**Figure 1:**
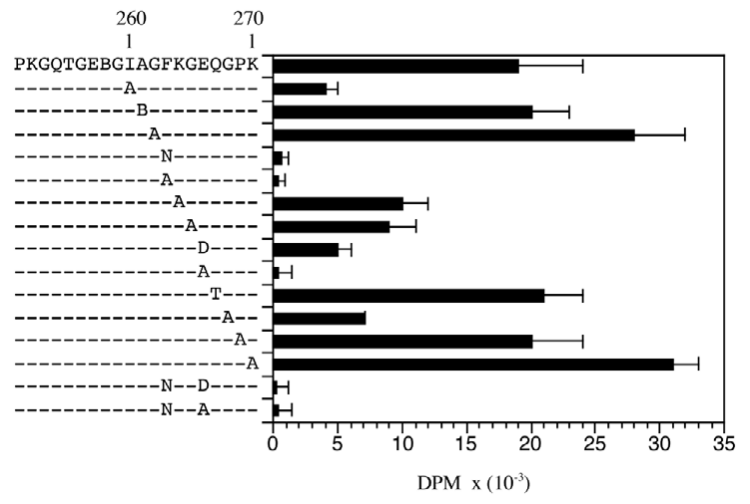
Proliferation of DR4-(DRB1*0401)-restricted T cells in response to type II collagen. A panel of analog peptides containing amino acid substitutions was tested for the ability to stimulate the proliferation of type II collagen (CII)-primed T cells. DR4 transgenic mice were immunized with CII; 10 days later, draining lymph node cells were harvested and cultured with the analog peptides. Proliferation was measured by [^3^H]thymidine incorporation and results are shown as means ± standard deviation (*x *axis). Data shown are from a representative experiment. Lymph node cells from six immunized mice were pooled before culture, and the data were subsequently confirmed in three separate experiments. Control cells cultured with no antigen had a proliferation value of 0.38 × 10^-3 ^d.p.m.

To select altered peptide ligands that might suppress arthritis, we used a panel of analog peptides containing substitutions at each position of the immunodominant DR4-restricted determinant of CII, replacing the wild-type residue with either alanine or the analogous amino acid present in type I collagen as we have done previously with DR1 molecules. The peptides were tested for reaction with T cells from DR4 transgenic mice that had been immunized with CII. Cells from draining lymph nodes of DR4 mice immunized with CII were cultured with each peptide, and the proliferative responses were measured with a [^3^H]thymidine incorporation assay. Variable T-cell responses were generated against the analog peptides (Figure 1). Substitutions of alanine for glycine at positions 265 and 268 did not seem to affect the response appreciably. However, the weakest responses were generated against peptides containing substitutions at positions 263 and 266. These results are comparable to those we have previously shown for DR1 and suggest that positions 263 and 266 in CII are critical for T-cell recognition of the peptide in the context of both DR1 and DR4 complexes.

### Effects of A12 and A13 on cytokine production by T cells

An important characteristic of an altered peptide ligand is its ability to alter the cytokine profile of responding T cells. We therefore determined the cytokine profile induced by analog peptides containing substitutions at residue 263 or 266 or both, with emphasis on IFN-γ, IL-10 and IL-4. When T cells from CII-immunized DR4 mice were cultured *in vitro *with various analog peptides (Table 1), the cytokine response to wild-type peptide was predominantly IFN-γ and IL-10. In contrast, analog peptides containing substitutions at residue 263 or 266 had a decreased IFN-γ response. The most effective combinations of both decreased IFN-γ and increased IL-4 responses were accomplished in analog peptides containing substitutions at both residues 263 and 266, namely A12 and A13. This combination of cytokine secretion could not be duplicated by low doses of the wild-type peptide. When CII-specific T cells were cultured with the wild-type peptide at low doses (10 μg/ml), secretion of IFN-γ was slightly above background (52 ± 5 pg/ml), whereas that of IL-4 decreased to undetectable levels.

Secretion of IL-4 is significant because it is effective in suppressing murine inflammatory arthritis [16-19]. Although analog peptides containing individual substitutions of F263N, E266D, and E266A decreased the IFN-γ response, they did not induce a significant IL-4 response. The combination of two substitutions at both residues 263 and 266 was required to produce both a decrease in IFN-γ and an increase in IL-4.

In a separate experiment, lymph nodes were taken from mice previously co-immunized with CII plus A12 or CII plus A13 and cultured with CII to determine whether the shift toward IL-4 could be confirmed *ex vivo*. Whereas the IFN-γ responses to CII were intermediate (425 ± 12 and 348 ± 14 pg/ml, respectively), the IL-4 responses were elevated (52 ± 8 and 49 ± 6 pg/ml, respectively).

### Binding of synthesized peptides to human DR4

**Figure 2:**
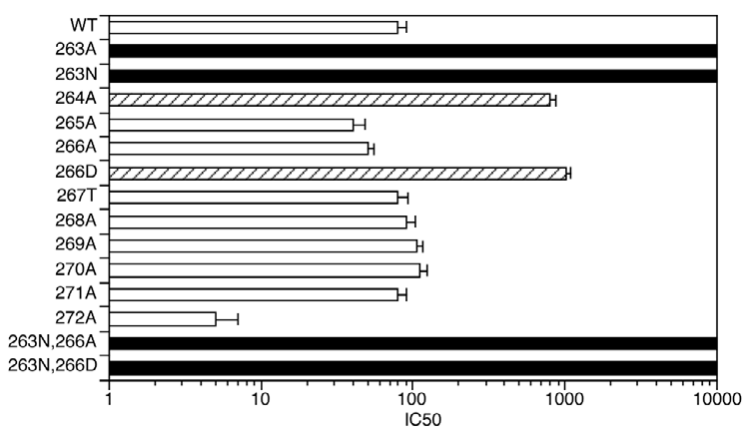
Binding of analog peptides to DRB1*0401. IC_50 _(50% inhibitory concentration) values are the average of two determinations per peptide. Peptides binding to DRB1*0401 with an IC_50 _of about 1,000 are represented by striped bars (264A, 266D). Peptides binding to DRB1*0401 with an IC_50 _of 100 or less (comparable to wild-type peptide binding) are represented by white bars.

T cells recognize peptides only if they are bound by major histocompatibility complex (MHC) molecules. Therefore, with the use of 21-mer synthesized peptides, DR-peptide binding assays were performed to determine the pattern of collagen binding to DR4. As shown in Figure 2, replacement of residue 263 with either asparagine or alanine significantly weakened binding. In contrast, replacement of residue 266 with an alanine or aspartic acid residue had differing effects. The peptide containing the small nonpolar alanine bound as effectively to DR4 as the wild-type peptide, whereas aspartic acid at position 266 caused a decrease in affinity with an IC_50 _of about 1,000. Peptides containing both substitutions, specifically A12 and A13, had significantly weaker affinities for DR4 than wild-type peptide (with IC_50 _values greater than 10,000). These data imply that competition with the wild-type peptide for binding to the DR4 is extremely unlikely as an etiology for the suppression of arthritis induced by A12 and A13. Moreover, substitutions at residue 266 of CII, which is the residue that interacts with the MHC shared epitope [20], cause wide variations in binding affinity.

### Effect of analog peptides on collagen-induced arthritis

**Figure 3:**
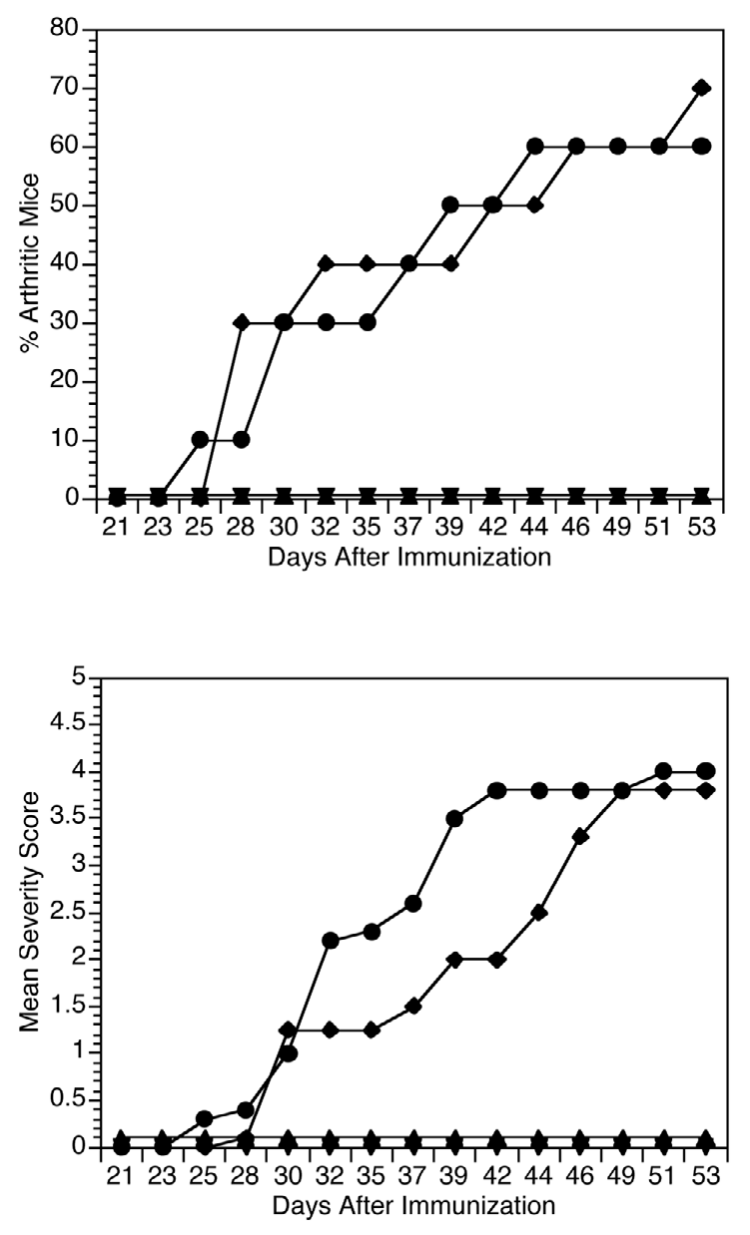
Co-immunization of DR4 transgenic mice with type II collagen and analog peptides. Groups of DR4 transgenic mice were co-immunized with 50 μg of type II collagen (CII) alone (*n *= 18; circles) or with 50 μg of CII plus 1.2 mg of the analog peptide CI_256–276_, (F263N, E266D) (*n *= 16; upward triangles), with 50 μg of CII plus 1.2 mg of the analog peptide CII_256–276 _(F263N, E266A) (*n *= 10; downward triangles), or with 50 μg of CII plus 1.2 mg of the analog peptide CII_256–276 _(E266D) (*n *= 10; diamonds) and observed for arthritis. Results are incidences (upper panel) and mean severity scores (lower panel) of arthritis observed at various time points after immunization.

Because both residues 263 and 266 of CII were identified as crucial for T-cell responses, analog peptides A12 and A13, containing substitutions at these two positions, were selected for testing in autoimmune arthritis *in vivo*. DR4 transgenic mice were immunized with CII, CII plus A12, CII plus A13, or CII plus the control peptide 266D, and were observed for the development of arthritis. When CII and either A12 or A13 were used at a molar ratio of 1:480, the development of arthritis was completely inhibited, whereas the control peptide had no effect. Concordant with a decrease in the incidence and severity of arthritis, the production of antibody against CII was also significantly decreased (Table 2 and Figure 3).

## Discussion

We report that A12, an analog peptide of type II collagen, is effective in suppressing autoimmune arthritis in mice bearing a transgene for HLA DRB1*0401. The finding that the same peptide is effective in both HLA DRB1*0401 and HLA DRB1*0101 is somewhat surprising but not totally unexpected because the immunodominant determinant, CII_256–276_, is common to both DR1 and DR4 transgenic mice. In addition to A12, we identified another altered peptide ligand, A13, containing substitutions within the immunodominant determinant of type II collagen that effectively suppress autoimmune arthritis in DR4 transgenic mice.

Both of these analog peptides contain substitutions at residues 263 and 266, which are anchor residues for DRB1*0401 [11,21-23]. It is evident that there is not a total absence of binding because both analogs can mediate a T-cell response, specifically an increased production of IL-4. However, the weak binding precludes either analog from outcompeting the wild-type peptide for binding to the MHC. In preliminary studies, if peptide A12 and wild-type peptide were covalently bound to soluble DR4 molecules in the presence of peptide HA and an HA-specific T hybrid, we found that A12 was more easily displaced than wild-type peptide by the HA, yet some displacement was required (EF Rosloniec, unpublished work).

The ability of A12 to suppress arthritis is correlated with a change in the cytokine response that included combinations of both decreased IFN-γ and increased IL-4 production. This suggests that the basis for suppression is the cytokine shift responsible for its activity. Interestingly, the individual collagen substitutions of F_263_→N, E_266_→D, and E_266_→A each decreased the IFN-γ response but did not induce a significant IL-4 response. The combination of two substitutions at both residues 263 and 266 was required to produce both a decrease in IFN-γ and an increase in IL-4. For DRB1*0401 the replacement of residue 266 with either D or A was effective, although the alanine seemed to be slightly more effective. The similarities between the two cytokine profiles were unexpected because the binding affinities vary significantly.

Crystal structure of HLA DRB1*0101 has revealed that the shared epitope at residues 70–74 forms part of the P4 binding pocket [20]. Although the shared epitope is similar for DR4 and DR1, DRB1*0101 has an arginine at residue 71, whereas DRB1*0401 has lysine. Crystal evidence suggests that residue 266 of CII interacts with DRB1 residue 71 [24], possibly explaining the haplotype differences we find when A13 is used to suppress arthritis. Although it has previously been thought that MHC binding was mostly independent of the MHC-peptide surface conformation, new technology with MHC-peptide tetramers reveals that changes in the residues interacting with the MHC binding pockets can induce subtle but important stereo chemical changes on residues positioned to interact with the TCR [25,26].

Our findings have implications for the treatment of RA. Susceptibility to RA is strongly associated with the expression of specific HLA class II alleles, and DRB1*0401 is the haplotype most commonly expressed in patients with RA [1,2]. DRB1*0101 is also associated with RA, although it is less common. T cells from patients with either allele are likely to have an altered response to A12. Treatment with this single peptide might therefore be effective for all RA susceptibility alleles that contain the 'shared epitope'.

## Conclusion

We have analyzed the DR-restricted presentation of the CII_256–276 _peptide and identified two analog peptides that suppress arthritis in the context of DRB1*0401. These experiments represent the first description of an analog peptide of type II collagen recognized by T cells in the context of the human DR4 molecule that can suppress autoimmune arthritis, and could become the basis for development of new therapies for RA.

## Abbreviations

BSA = bovine serum albumin; CFA = complete Freund's adjuvant; CIA = collagen-induced arthritis; CII = type II collagen; IC_50 _= 50% inhibitory concentration; IFN = interferon; IL = interleukin; MHC = major histocompatibility complex; OcG = octyl glucoside; RA = rheumatoid arthritis.

## Competing interests

The authors declare that they have no competing interests.

## Authors' contributions

YS, DDB and BT performed the experiments in this manuscript. EFR, JMS, AHK and LKM provided supervision and experimental design. All authors read and approved the final manuscript.

## Figures and Tables

**Table 1:** DR4 T-cell response to analog peptides after immunization with type II collagen (CII)

Peptide	Sequence^a 263 266^	IFN-γ (pg/ml)	IL-10 (pg/ml)	IL-4 (pg/ml)
Wild type	G-E-B-G-I-A-G-F-K-G-E-Q-G-P-K-G-E-B-G-P-A	1,575 ± 20	570 ± 8	12 ± 3
A9	- - - - A B - N - - - - - - - - - - - - -	350 ± 11	580 ± 11	**33 ± 12**
A12	- - - - - - - N - - D - - - - - - - - - -	175 ± 7	610 ± 7	**48 ± 15**
A13	- - - - - - - N - - A - - - - - - - - - -	<1	575 ± 9	**66 ± 10**
263N	- - - - - - - N - - - - - - - - - - - - -	263 ± 13	560 ± 13	9 ± 9
266A	- - - - - - - - - - A - - - - - - - - - -	<1	590 ± 10	6 ± 9
266D	- - - - - - - - - - D - - - - - - - - - -	175 ± 6	600 ± 12	9 ± 5

Pooled splenocytes and lymph node cells (5 × 10^6 ^cells/ml) from DR4 mice immunized with CII 10 to 14 days previously were cultured with 100 μg/ml antigen (synthetic peptides). Each experiment included cells from three mice and was performed three times. Supernatants were collected and assayed for IFN-γ, IL-4, and IL-10. Values are means ± SD. Numbers in bold type indicate values greater than fivefold those of wild-type peptide. The cytokine response to wild-type peptide included primarily IFN-γ and IL-10. In contrast, the analog peptides A12 and A13 induced the production of predominantly IL-4 and IL-10.

**Table 2:** Co-immunization of DR4 transgenic mice with type II collagen (CII) and analog peptides

Immunizing antigen	Core of co-immunogen	Incidence of arthritis	CII antibodies
CII alone	FKGEQGPKG	11/18 (61%)	61 ± 11
CII + A13 (1:480)	N--A-----	0/10^a^	7 ± 2^b^
CII + A12 (1:480)	N--D-----	0/16^b^	16 ± 9^b^
CII + D266 (1:480)	---D-----	7/10 (70%)	48 ± 13

Groups of DR4 transgenic mice were co-immunized with CII alone or CII plus an analog peptide at the molar ratios indicated, and were observed for arthritis. These doses were based on dose-response curves obtained using DR1 mice [12]. Results shown represent the incidence of arthritis observed at 8 weeks after immunization. Mice were bled at 6 weeks after immunization, and sera were analyzed individually by enzyme-linked immunosorbent assay to determine antibody levels against bovine CII. Results are means ± SD for each group of animals and are expressed in units of activity. ^a^*p *≤ 0.01; ^b^*p *≤ 0.001.
